# Variance Heterogeneity in *Saccharomyces cerevisiae* Expression Data: Trans-Regulation and Epistasis 

**DOI:** 10.1371/journal.pone.0079507

**Published:** 2013-11-04

**Authors:** Ronald M. Nelson, Mats E. Pettersson, Xidan Li, Örjan Carlborg

**Affiliations:** Department of Clinical Sciences, Swedish University of Agricultural Sciences, Uppsala, Sweden; National Institute of Environmental and Health Sciences, United States of America

## Abstract

Here, we describe the results from the first variance heterogeneity Genome Wide Association Study (VGWAS) on yeast expression data. Using this forward genetics approach, we show that the genetic regulation of gene-expression in the budding yeast, *Saccharomyces cerevisiae*, includes mechanisms that can lead to variance heterogeneity in the expression between genotypes. Additionally, we performed a mean effect association study (GWAS). Comparing the mean and variance heterogeneity analyses, we find that the mean expression level is under genetic regulation from a larger absolute number of loci but that a higher proportion of the variance controlling loci were trans-regulated. Both mean and variance regulating loci cluster in regulatory hotspots that affect a large number of phenotypes; a single variance-controlling locus, mapping close to *DIA2*, was found to be involved in more than 10% of the significant associations. It has been suggested in the literature that variance-heterogeneity between the genotypes might be due to genetic interactions. We therefore screened the multi-locus genotype-phenotype maps for several traits where multiple associations were found, for indications of epistasis. Several examples of two and three locus genetic interactions were found to involve variance-controlling loci, with reports from the literature corroborating the functional connections between the loci. By using a new analytical approach to re-analyze a powerful existing dataset, we are thus able to both provide novel insights to the genetic mechanisms involved in the regulation of gene-expression in budding yeast and experimentally validate epistasis as an important mechanism underlying genetic variance-heterogeneity between genotypes.

## Introduction

Unravelling the genetic architecture of complex traits is a grand challenge in current biology. New tools such as dense genetic maps, high-throughput genotyping and large-scale phenomics have made genome-wide association studies (GWAS) a widely applied approach. The detected genes do, however, often explain only a small amount of the phenotypic variability. A potential reason could be that the contribution of complex interactions of multiple genes is common [1,2], while the most frequently used analysis methods assume that inheritance is purely additive. There is however an increase in reports on variance due to genetic interactions [3,4]. In general, the contribution to trait variability of interacting loci is difficult to assess as only the strongest interactions can be detected due to the high significance thresholds needed to correct for the multiple-testing performed in multi-dimensional scans for interacting loci. For a more complete exploration, new methods are needed that allow detection of all types of epistatic interactions, i.e. also those that do not lead to strong main effects, without using excessive multiple testing. Furthermore, a more extensive use of model organisms for this purpose would also be beneficial as the underlying biology of epistatic interactions is difficult to assess in studies based on complex phenotypic data in higher organisms. Thus, a more widespread use of new methods for detecting genetic interactions to analyze relatively simple phenotypes in well-studied model organisms has the potential to uncover more information on the biology and genetics underlying the complex genetic interactions. In this way we can guide future efforts to dissect important agricultural and medical traits in higher organisms.

An example where this approach has earlier been used is for the genetic dissection of the genetic regulation of gene expression in *Saccharomyces cerevisiae*. By crossing divergent strains and analysing data on genotypes and phenotypes in multiple haploid segregants, a large number of genes, genomic regions and complex networks involved in regulating expression have been found [5-11].

Although complex to measure and analyze in itself, expression data can be considered as a relatively simple genetic trait, since the regulation of a single gene’s expression is expected to involve much fewer factors than more complex phenotypes such as growth rate, behaviour and disease susceptibility. Furthermore, by using expression data as a model phenotype for testing analysis methods for finding interactions, it will not only allow an estimate of the total contribution of epistasis to the trait, but also allow researchers to utilize the wealth of available knowledge of yeast biology [12]to functionally explain the inferred interactions. 

The variance-heterogeneity GWAS, or vGWAS for short [13-15], is a method developed to detect genomic loci that display variance heterogeneity between genotypes. This heterogeneity could be due to several underlying mechanisms, one of them being epistasis. The reason for this is that, when there is non-additive interaction between two loci, variance heterogeneity is introduced into at least one locus when a one-dimensional search is performed [15,16]. This means that the loci detected in the vGWAS are likely candidates for being involved in gene-by-environment or gene-by-gene interactions [13-15,17]. As vGWAS is based on a one-dimensional scan of the genome, it reduces the multiple testing penalty in screens for epistatic interactions by identifying a smaller subset of candidate interacting loci among which gene-gene interactions can be detected. The method therefore has the potential to both improve power, due to a decreased need for multiple-testing corrections, and to considerably decrease the computational demand of multi-locus analyses. 

Variance heterogeneity has been observed in a number of studies [18-21] and has also been implicated in complex genome wide regulation of gene by RNA degradation in humans [22]. It has also been shown that vGWAS is able to identify many novel variance controlling loci and that they can make large contributions to the phenotypic variance of many complex traits in *Arabidopsis thaliana* [15]. In a separate study, the physiological and metabolic stochastic noise observed in *Arabidopsis thaliana* have been shown to be under genetic control [23]. The underlying biology is, however, largely unknown and it is therefore of interest to further explore the mechanisms underlying such effects in other experimental systems. 

Here, we apply both GWAS and vGWAS, analyses on data from a publically available *Saccharomyces cerevisiae* dataset [9] to find QTL and variance controlling QTL (vQTL) regulating gene-expression. Although this dataset has been well studied before [5,6,9], we find many previously unidentified loci involved in regulation of expression and highlight a few cases where vQTL are involved in epistatic interactions. We focus on gene-gene interactions, since the yeast model is well studied and incorporating database information, supporting functional links between genes, allows us to present a comprehensive description of the genetic architecture. 

Furthermore, we report a description of the distribution of the QTL and vQTL in the genome and discuss the potential functional relevance of the overlap of the loci in hotspots. In light of this analysis, vGWAS emerges as a promising approach to functionally dissect the complex genetics underlying gene-expression. By combining the results from GWAS and vGWAS analyses with available knowledge on biological networks, we connect the results with known molecular mechanisms in yeast, verify established pathways and, more importantly, provide a new route to more complete dissection of the genetics of complex traits.

## Results and Discussion

We have mapped genes affecting either the mean difference or variance heterogeneity in expression levels of single genes in a cross between two divergent yeast strains. We describe the genomic distribution of variance controlling loci in the genome, compare it to the distribution of mean controlling loci and are able to identify potential master loci involved in the regulation of genome-wide variance heterogeneity in expression. By exploring whether genetic interactions is a plausible explanation for the observed genetic variance heterogeneity between genotypes at the vQTL, we provide new insights to their role in multi-locus interactions. 

### Genome-wide mapping of QTL and vQTL

In total, 8387 QTL and vQTL were identified for the 4482 expression phenotypes across the two treatments (Table 1). Study-wide, there are considerably more QTL than vQTL in the genome (8196 vs. 191) with a relatively even distribution of signals across the two treatments. The total number of vQTL is thus lower in this study than in some of the other earlier studies of more complex phenotypes in higher organisms [15]. This is likely due to the fact that expression phenotypes are, by nature, less genetically complex than phenotypes measured on an organismal level (e.g. weight, growth rate or flowering time), the number of gene-gene (epistatic) interactions that could lead to a variance heterogeneity between genotypes are lower than for macroscopic traits. On the other hand, it is expected that the vQTL affecting the expression phenotypes will have a tight connection with the biology of the particular trait and therefore more useful for dissecting the underlying mechanisms of the signals. The results from this study conform to the expectation that less complex phenotypes are affected by fewer loci since, a study on more complex phenotypes observed equal numbers of QTL and vQTL [15].

**Table 1:** The number of significant QTL and vQTL peaks per treatment.

	**Glucose**			**Ethanol**			**Proportion trans**
	**cis**	**trans**	**Total^a^**	**cis**	**trans**	**Total^a^**	
QTL	1541	2845	4397	1563	2228	3799	0.62
vQTL	13	95	108	8	75	83	0.89
Total	1554	2940	4505	1571	2303	3882	

SNP peaks separated by 50,000bp were considered to be two peaks.

^a^ A few expression phenotypes have unknown genome positions and thus the total number of QTL is larger than the sum of the cis and trans QTL.

**Figure 1:**
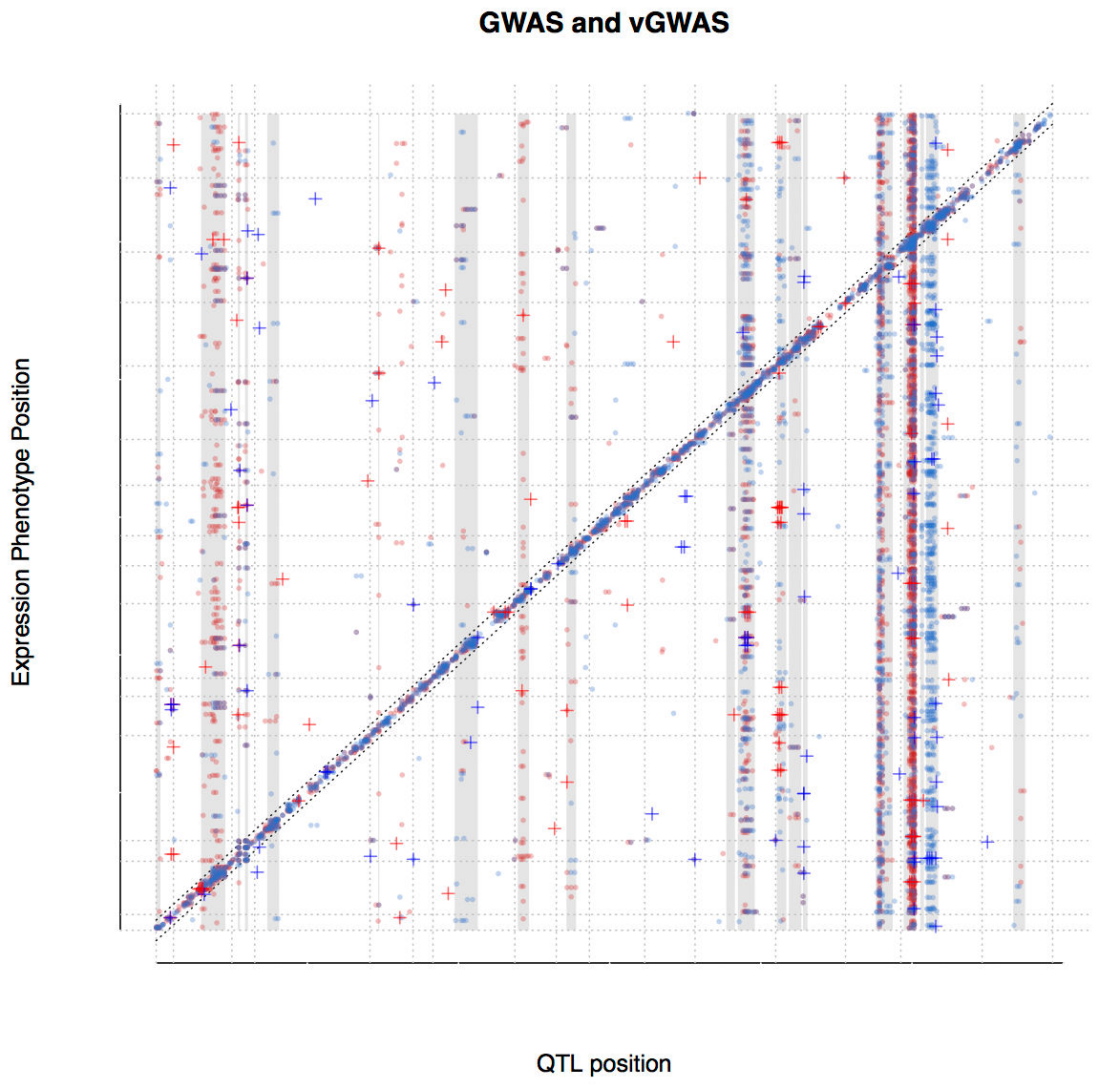
Genome wide distribution of QTL and vQTL. The distribution of QTL and vQTL with significant effects on gene-expression across the yeast genome (for a by-chromosome version see Figure S1). Red and blue indicate significant QTL/vQTL peaks for glucose and ethanol treatments respectively. Closed circles indicate QTL affecting the mean level of expression and crosses vQTL affecting the variance heterogeneity. Chromosome boundaries are indicated by gray, horizontal and vertical lines. Cis-QTL are located on the diagonal with our defined cis-/trans-border indicated black dotted lines (Table 1). The shaded vertical bands containing many peaks are suggested hotspots of gene-regulation (see Figure 6B).

The genome-wide distribution of QTL and vQTL in cis- and trans- was visualized by plotting the location of the QTL and vQTL peaks across all phenotypes against the position of the expression phenotype they affect (Figure 1; Figure S1; Tables S1, S2). Roughly 25% of the peaks fall close to the diagonal in Figure 1, and are thus cis-QTL or cis-vQTL, respectively. In Table 1, we summarize the locations of the sets of significant QTL and vQTL relative to the expression phenotype they affect. There is slightly less than two times as many trans-QTL as cis-QTL, while, the trans-vQTL are eight times more abundant than the cis-vQTL. 

**Figure 2:**
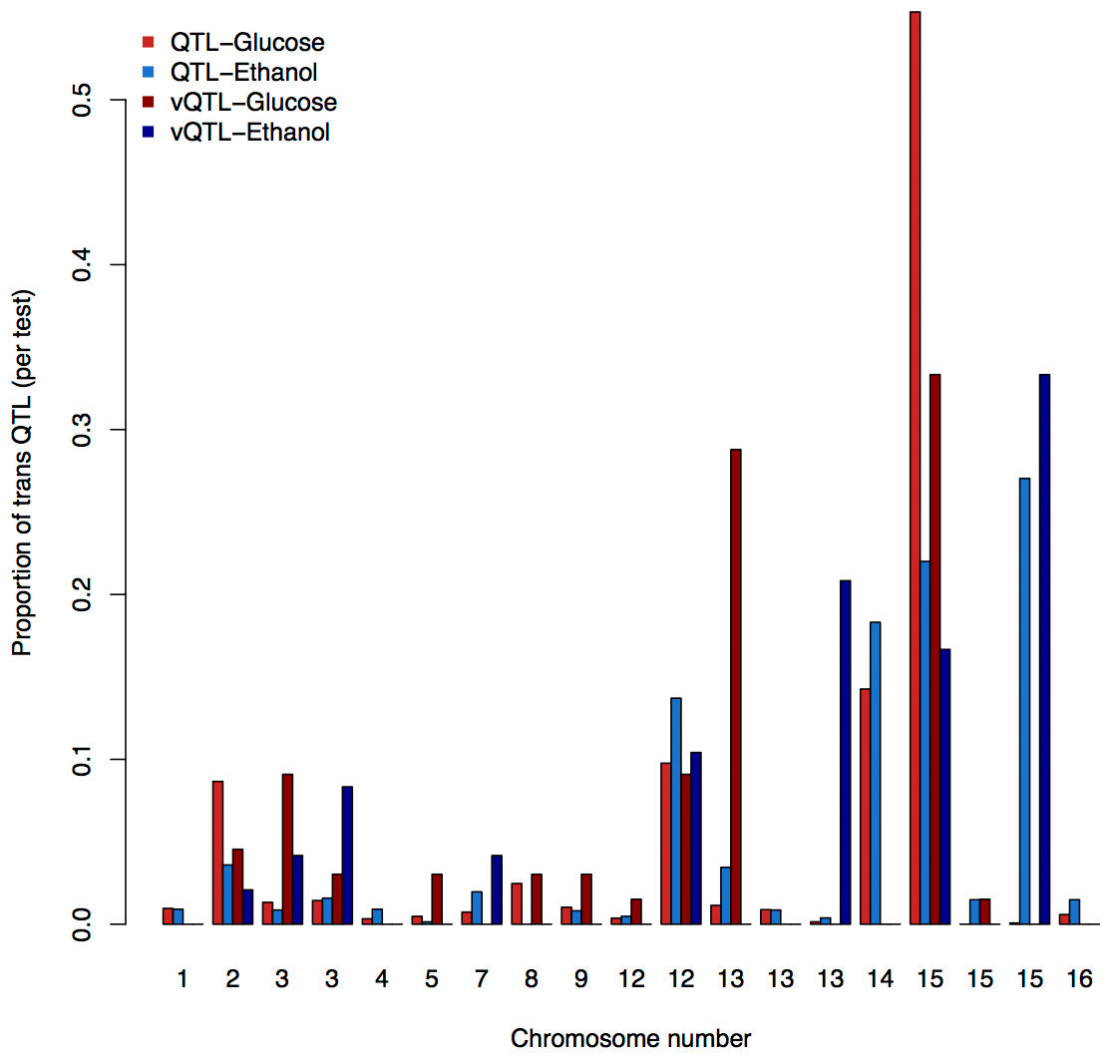
Distribution of QTL and vQTL across hotspots. A comparison of all proportion QTL and vQTL for each treatment for the 19 gene-regulation hotstpots in the yeast genome (see also Table S1).

Figure 1 shows that a number of QTL or vQTL in the genome affect multiple phenotypes. These hotspots, that together regulate transcription of thousands of genes distributed throughout the genome, are themselves spread across the genome and have earlier been reported in this data (Figure 1, Figure S1; see 5,9). In Table 2, we summarize the locations of the significant trans-QTL/vQTL relative to the 19 visually identified hotspots, which in total cover approximately 22% the genome. There is a significant bias for the trans-QTL/vQTL to be located within a hotspot (Table S3). The majority of the trans-QTL (95%) and trans-vQTL (67%) fall within one of these hotspot regions (Figure 2; Table S1). More than 40 percent of all the trans-QTL/vQTL are located in the first hotspot on chromosome 15. Several of the hotspots include vQTL and some are biased in favor of vQTL (Figure 2).

**Table 2:** Distribution of trans QTL and vQTL across the genome (see also Table S1).

	**Glucose - trans**			**Ethanol - trans**			**Proportion in hotspot**
	**Hotspot**	**Non-hotspot**	**Total**	**Hotspot**	**Non-hotspot**	**Total**	
QTL	2713	132	2845	2086	142	2228	0.95
vQTL	66	29	95	48	27	75	0.67
Total	2779	161	2940	2134	169	2303	

Trans acting QTL are found to regulate approximately 60% of the expression phenotypes, as also reported earlier [5,6,9]. However we find that almost 90% of the vQTL are in trans relative to the expression phenotypes they affect. Although the total number of trans-QTL is very large (4941), only 274 of these are located outside of a hotspot. This is in contrast to the trans-vQTL, where 56 out of 170 are located outside of the hotspots. There are thus proportionally more trans-vQTL than trans-QTL and there are significantly more trans-QTL than trans-vQTL in hotspots (Table S3). This is consistent with the expectation that vQTL are more likely to be involved in epistatic interactions and act as key regulatory elements in particular biochemical pathways, whereas the expression QTL are expected to be master regulators that act simultaneously on many other loci. 

The distribution of the cis- and trans-vQTL is consistent with the fact that epistatic regulation of gene-expression will, by definition, include trans-effects. A number of vQTL are located in hotspot regions, indicating that epistasis might also involve some master-regulators. Hotspot 18, on chromosome 15 (Table S1), may be one such regulator, containing almost 10% of all the vQTL. The gene *DIA2* is located within this hotspot and has been indicated as a gene essential for genome stability [24]. It is thus a potential candidate gene for introducing high levels of variance heterogeneity between functional and disrupted alleles at many loci across the genome, potentially by causing high levels of chromosomal rearrangements [24].

Most vQTL are, however, located outside of the hotspots, presumably as part of more specific, epistatic regulatory control systems. Although the number of vQTL inferred here is small relative to the number of QTL (only about 2% of the total number of significant loci), they represent about 20% of the regulatory loci that are neither master-regulators nor auto-regulatory. The picture thus emerges that epistasis might be an important factor in specific regulatory control of individual genes and that identification of vQTL emerges as a useful approach to identify such novel regulatory elements in the genome.

### The expression-traits affected by vQTL

In total, 133 phenotypes had at least one significant vQTL (61 in glucose-, 72 in ethanol-, and 8 in both treatments; Table S2). To explore whether some of the vQTL were strong candidates for being involved in epistatic interactions, we mined the results for potential interactions between the vQTL, the gene whose expression it affects and other vQTL or QTL affecting the same expression phenotype. Unbalanced sampling in the genotypic classes may inflate the significance of the variance (Shen and Carlborg submitted), however, the vQTL reported here have balanced genotype frequencies due to the F_2_ design, this artefact is thus avoided (Shen and Carlborg submitted). The first step in the evaluation was to inspect the pairwise genotype-phenotype (GP) maps for all possible pairs of loci affecting a given phenotype for signs of epistasis. If the GP-map indicated epistasis, all genes in the region ±10kb of each QTL and vQTL peaks were identified and a search conducted in the literature and relevant databases for known interactions between the genes in the epistatic regions (Table S4). In the following sections we will discuss four illustrative examples of two- and three-locus interactions involving vQTL as a way to illustrate the type of effects that could be detected by vGWAS analyses.

### Epistatic interactions in the regulation of flocculation

**Figure 3:**
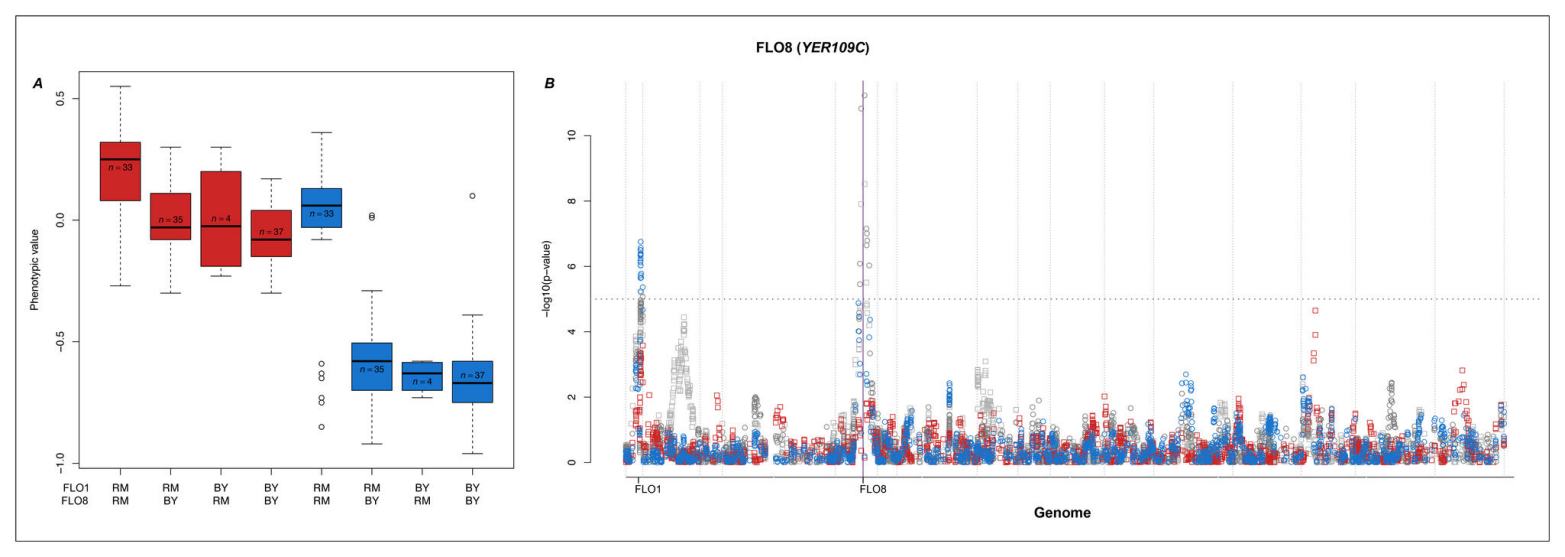
QTL and vQTL regulation of *FLO8*. Using a GWAS and vGWAS analysis, several loci were identified as either QTL, vQTL or both in their effects on gene-expression of *FLO8*. *A*) The genotype-phenotype map for the glucose (red) and ethanol (blue) treatments for genotypic combinations of all the QTL and vQTL identified in *B*. *B*) Manhattan-plots for GWAS (grey) and vGWAS (coloured). Squares and circles represent markers from the glucose and ethanol treatments respectively. The horizontal dotted line represents 0.5% FDR significance level. The purple vertical line indicates the position of the expression phenotype.

The expression of the flocculation gene *FLO8* (YER109C; Figure 3) is regulated by multiple loci. The mean expression level is regulated by a cis-QTL located 16kb from the gene itself. A trans-vQTL on Chromosome 1 (200,997bp, Figure 3B) shifts the mean expression level and also introduces a genetic variance-heterogeneity in the expression of *FLO8*. The genotype-phenotype map for the interaction between these two loci showed a distinct epistatic pattern in the ethanol treatment (Figure 3A), where there is up-regulation of expression of *FLO8* if and only if there are RM-derived alleles at both loci. In all other genotypic combinations, expression is lower. The genes involved in flocculation have been well studied [25-27] and both of the regions identified in this study contain candidate genes for the observed effects. The most obvious candidate gene in the region of the cis-QTL is *FLO8* itself and the peak on Chromosome 1 contains the gene *FLO1* (YAR050W; 203,403-208,016 bp). The interaction between *FLO1* and *FLO8* and its role in flocculation has been experimentally verified [25-27] and it is known that the expression of *FLO8* regulates the expression of *FLO1* [25,26]. The link between *FLO1* and *FLO8* has earlier been reported in this population [5], but our vQTL results provide additional insights to the nature of this interaction by showing that the expression, and especially the variance in the expression, of *FLO8* is affected by *FLO1*. Although the genes in this particular example can be detected also in a GWAS for mean effects, it illustrates the ability of the vGWAS to not only identify QTL but also indicate potential epistatic interactions to be explored in more detail. 

### Epistatic interactions involving multiple loci in the pyrimidine metabolism pathway

**Figure 4:**
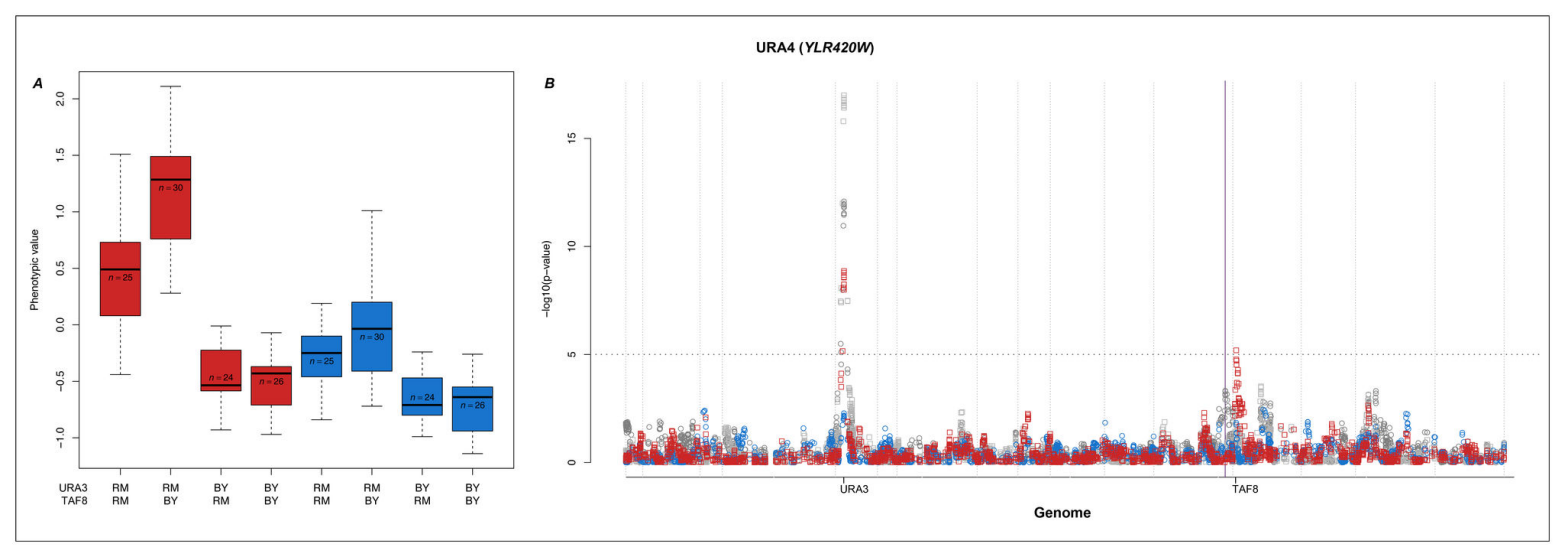
QTL and vQTL regulation of *URA4*. A) The genotype-phenotype map of *URA4* expression for the two-way interactions identified in B (colours are the same as Figure 3). B) Manhattan-plots of GWAS and vGWAS of the *URA4* phenotype. The horizontal dotted line represents 0.5% FDR significance level. The purple vertical line indicates the position of the expression phenotype. Colours and symbols are the same as Figure 3.

Several of the identified vQTL are involved in two-way interactions in the pyrmidine metabolism pathway (Figure 4). The expression of *URA4* (YLR420W) is affected by two variance-controlling loci when grown in glucose. The first is located in a hotspot on Chromosome 5 (117,705bp; Figure 4B; hotspot 6 Table S1) and causes both a shift in the mean expression level and variance heterogeneity in *URA4* expression. The second locus, located on Chromosome 13 (46,084bp; Figure 4B), only introduces a variance-heterogeneity between the alternative genotypes. The two-locus genotype-phenotype map (Figure 4A) shows a clear epistatic interaction between the two vQTL: the expression of *URA4* is up-regulated only in the RM genotype background for the vQTL on Chromosome 5, but expression is, on average much higher in the BY genotype background for the vQTL on chromosome 13. The genetic effects on *URA4* expression in the ethanol treatment are smaller and non-significant, but follow the same general trend.

The literature and database mining provides a promising candidate gene for the vQTL effect on Chromosome 5: *URA3* (YEL021W; 116,167-116,970bp). Both *URA3* and *URA4* are enzymes in the pyrimidine metabolism pathway. *URA3* is downstream of *URA4* with three intermediate metabolites between them (for a review on the topic see [28]). There is, however, no known direct links between *URA3* and *URA4*, but the results obtained here indicate that it should be worthwhile to further explore the functional link between these loci by e.g. looking for a potential feed-back or feed-forward mechanism involving these two loci.

Several candidate genes are located in the vQTL region on Chromosome 13, (Table S4), the most interesting being *TAF8* (YML114C) that, like *URA3* and *URA4*, also effects pyrimidine metabolism through the transcription factor *PPR1*. *PPR1* (YLR014C) is a key regulator in the activation of the genes in the pyrimidine pathway, especially during pyrimidine starvation and it interacts with other proteins in the transcription machinery during pyrimidine biosynthesis [28,29]. *TAF8* is a transcription factor subunit with a verified genetic link with *PPR1* [30]. The promoter sites of *URA3* and *URA4*, where *PPR1* binds, are similar and there is thus competitive binding [31]. *URA4* is only up regulated when the QTL at *URA3* is from the RM strain and it is further enhanced when the vQTL close to the *TAF8* gene is from the BY strain. Further experimental work is needed to explore the molecular mechanisms underlying this epistatic interaction. It would e.g. be interesting to test whether there is differential binding affinity of *PPR1* to the *URA3/URA4* promoters when it is combined with the alternative TAF8 alleles and whether leads to a differential expression of *URA3* and/or *URA4* that could explain the differences observed in expression of *URA4* in this study. 

### Multi-locus interactions affecting the expression of CTA1 (YDR256C)

**Figure 5:**
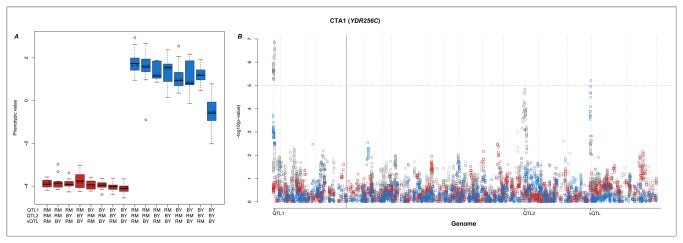
QTL and vQTL regulation of *CTA1*. A) The genotype-phenotype map of *CTA1* expression for the three interactions identified in B (colours are the same as Figure 3). B) Manhattan-plots of GWAS and vGWAS of the *CTA1* phenotype. The horizontal dotted line represents 0.5% FDR significance level. The purple vertical line indicates the position of the expression phenotype. Colours and symbols are the same as Figure 3.

The expression of *CTA1* in the ethanol treatment is affected by three loci (Figure 5). The first two are trans-QTL in hotspots on chromosome 1 (42,489bp; hotspot 1, Table S1) and on chromosome 12 (662,627bp; hotspot 11, Table S1), while the third is a vQTL on chromosome 14 (714,215bp). The three-locus genotype-phenotype map (Figure 5A) illustrates a clear three-locus epistatic interaction, where individuals with the BY genotype at all three loci have down regulated expression of *CTA1*. Any other genotypic combination results in up regulation of *CTA1*; an example of a classic three-locus, duplicate-factor epistasis. 

*CTA1* encodes a catalase enzyme that decomposes hydrogen peroxide in the perisomes and mitochondria during fatty acid beta oxidation [32]. Its expression is increased during caloric restriction and oxidative stress, as is the case in exposure to ethanol [33]. The expression of *CTA1* is lower in individuals with the BY genotype at all three loci, i.e. in the domesticated genotype. An explanation for this pattern is that these loci are involved in making the domesticated strain better adapted to deal with exposure to the oxidative stress under the ethanol treatment, possibly due to multiple exposures in during domestication and use in fermentation. Its response to this exposure would therefore not be as strong. The identification of the vQTL, in addition to the two QTL, allowed this three-way interaction to be detected. However, although a number of candidate genes could be identified in the QTL and vQTL regions (Table S4), we were not able to assign any of these to a coherent network that has been experimentally verified before. Further investigation of the candidate genes (Table S4) would be interesting as it may reveal genes and biochemical mechanism responsible for response to oxidative stress through multi-locus epistatic regulation of expression. 

### An interaction involving DIA2 (YOR080W) and Rim8 controls the variance-heterogeneity in expression of VPS20 (YMR077C)

**Figure 6:**
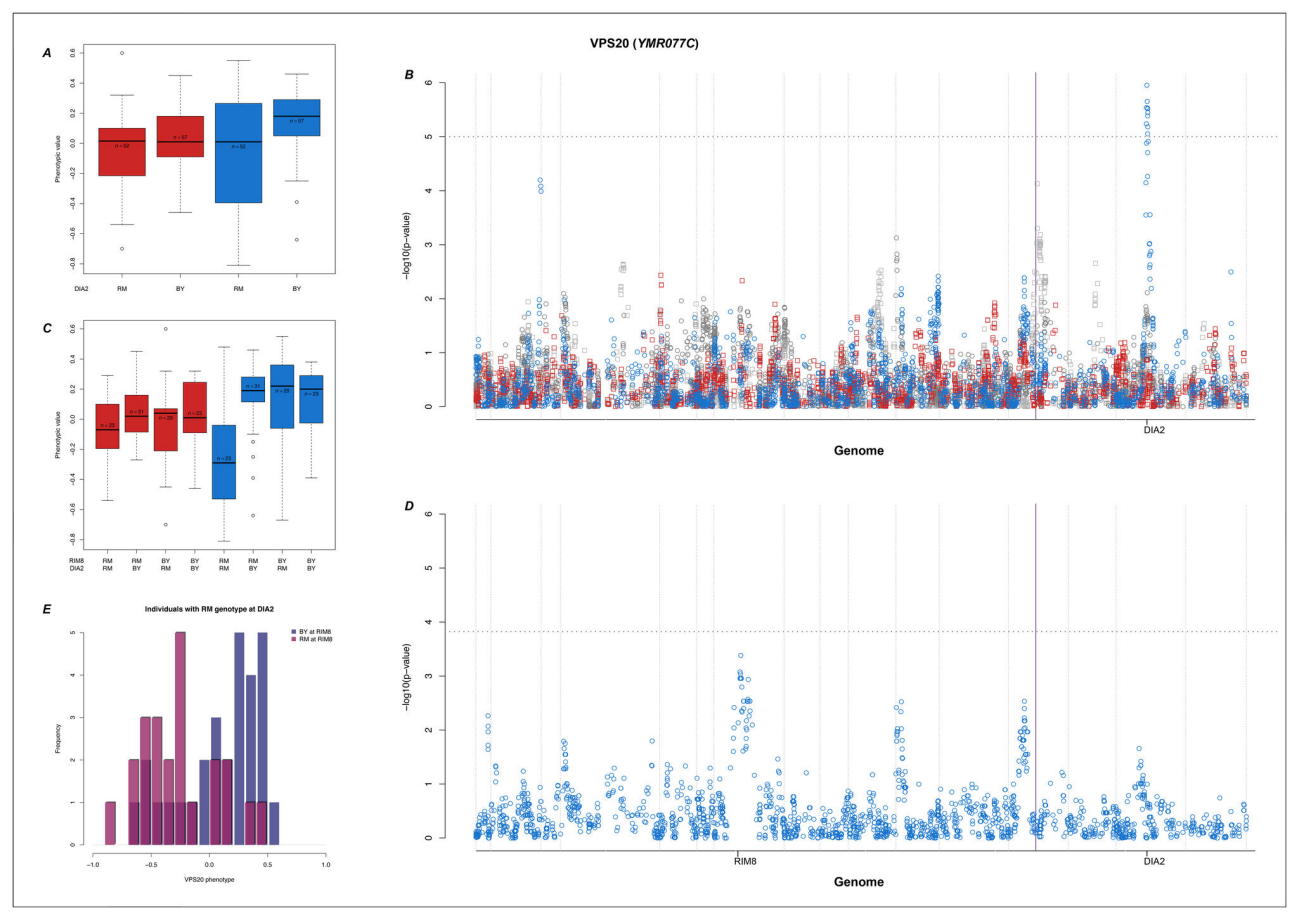
QTL and vQTL regulation of *VPS20*. A) Expression of *VPS20* for the different genotypes at the *DIA2* locus (colours are the same as Figure 3). B) Manhattan-plots of GWAS and vGWAS of the *VPS20* phenotype. The horizontal dotted line represents 0.5% FDR significance level. The purple vertical line indicates the position of the expression phenotype. Colours and symbols are the same as Figure 3. C) The genotype-phenotype map of *VPS20* expression for the two way interactions identified from the vGWAS B. and GWAS D. D) Manhattan-plots of GWAS on *VPS20* expression in individuals with RM genotype at the *DIA2* locus. The horizontal dotted line represents 5% FDR significance level. E) Distribution of *VPS20* expression levels for individuals with RM genotype at *DIA2*.

In the ethanol treatment, we detect a significant genetic variance-heterogeneity for the expression of *VPS20* between the alternative genotypes of a vQTL on chromosome 15 (476,328 bp) (Figure 6A and 6B). This trans-vQTL is located in a hotspot on chromosome 15 (hotspot 18, Table S1) within the *DIA2* gene that contains almost 12% of the significant trans-QTL/vQTL for all the expression phenotypes. *DIA2* is known to play a central and regulatory role in a number of mini-pathways [34], and has been implicated in genome stability [24] affecting a number phenotypes and gene expressions. The genetic link between *DIA2* and *VPS20* is confirmed [34]. Both *VPS20* and *DIA2* are involved in ubiquitin-dependent protein degradation where *VPS20* interacts with other subunits to form complexes involved in transportation and localization of proteins in the degradation pathway [35-39]. Upon further inspection, the distribution of the *VPS20* expression within the high-variance *DIA2* RM genotype appears to be bimodal (Figure 6E). In an attempt to identify the reason for this bimodality, a second association analysis was performed where only individuals that had the RM genotype at the chromosome 15 vQTL were included (Figure 6D and 6E). The strongest signal in this analysis maps to the gene *RIM8* (YGL045W). This is an interesting finding as *RIM8* has earlier been reported to interact with *RIM101* (YHL027W; see 37,40) which in turn interacts with both *VPS20* and *DIA2* [30,37,41]. All these genes are involved in the ESCRT pathway, and, more specifically, transportation and interaction with ubiquitin modified proteins [30,35-39,41].

## Conclusions

When variance-heterogeneity exists between genotypes at a locus, it is an indication that the locus could be involved in epistatic interactions with other loci. When testing for epistatic QTL, a vQTL scan combined with a conditional mean effect scan has been shown to be more powerful than a two dimensional mean effect QTL scan [15]. Here we show evidence, indicating that several such vQTL are involved in the regulation of gene-expression. vQTL were less common than QTL affecting the mean, rarely acted in cis-, but constituted a significant portion of the trans-QTL, in particular trans-QTL located outside of suggested master regulatory regions (hotspots). This observation is in line with the expectation that vQTL are more likely to be involved in epistatic interactions modifying the expression in particular systems rather than act as master regulators of expression. One exception to this pattern is a hotspot on chromosome 15 close to the *DIA2* gene that contains 12% of the trans-QTL/vQTL and therefore might be a master regulator of genome-wide variance heterogeneity. 

 In this study, we provide several examples of interactions involving vQTL where links between candidate genes have been described before and in this way add new insights to how these links might be functionally connected through genetic interactions. In this way it is shown that the additional information gained from vGWAS analysis may provide useful additional insight into how loci combine to regulate expression and also contribute to the variation observed in the phenotype of the studied organism. 

## Methods

### Data

We re-analysed a publically available yeast dataset with genotypes and expression phenotypes on individuals from cross between a laboratory *S. cereviciae* strain (BY4716 , isogenic to S288C) and a wild isolate (RM11-1a)(for a complete description, see [7, 9]). Briefly, the dataset consisted of 109 haploid segregants. Each segregant was grown in two conditions with either glucose or ethanol as the main carbon energy source. Bar the different growth mediums, all other environmental conditions were kept constant throughout the experiment. For each segregant, a set of 2956 SNP markers were genotyped providing an average marker density of one marker every 4.1 kbp. The expression profiles for 4482 genes were obtained for each segregant. The expression profile of each individual consisted of the normalized and log_2_-transfomed hybridization profiles of RNA, from the 4482 genes, to cDNA microarrays (for each treatment). These were used as the phenotypes in the GWAS and vGWAS. 

### Genome wide association analysis for mean controlling loci (GWAS)

For each of the expression phenotypes, a genome-wide association analysis to detect mean effects of loci was performed using the Wilcoxon test to test for association at all genotyped marker locations. The Wilcoxon rank-sum test is non-parametric and is a standard test used for association studies. The analyses were performed in R, using a modified verions of the vGWAS package [15] with the Wilcoxon test replacing the variance-comparing Brown-Forsyth test. Significance was estimated using the procedure below.

### Genome-wide association analysis for variance controlling loci (vGWAS)

For each expression phenotype, a genome-wide association analysis to detect variance controlling loci was performed, using the Brown-Forsythe test implemented in the vGWAS package in R [15]. The Brown-Forsythe test assesses the equality of variances between different samples. The application to genomic data, as in the vGWAS package, means that the spread of the variances of the phenotype given the genotype is tested. Loci affecting the variance in expression are detected using this test [13-15]. Significance was estimated using the procedure below.

### Significance thresholds

The significance for both the GWAS and vGWAS was obtained using a method similar to that used in Smith and Kruglyak [9]. Permutation testing was performed on two hundred random phenotypes with one hundred permutations for each phenotype, resulting in a combined total of 20000 permutations. This was repeated for each of the treatments for both GWAS and vGWAS (i.e. four separate permutation test sets). Random phenotypes were used to ensure there was no systematic bias due to a specific phenotype. For each permutation the values of the selected phenotype was randomly assigned to the genotypes. Assuming that there is no true signal in the randomly assigned phenotypes, we obtained a distribution of false positive signals. Based on this distribution, we chose a threshold of 1.00x10^-5^. For the four permutation test sets, this threshold corresponds to a FDR of ranging from 0.43% to 0.48%.

In the *VPS20* example specifically, we identified a vQTL peak close to the *DIA2* gene (Figure 6A and 6B) using the thresholds calculated above. A bimodal distribution is seen in the phenotypes for the individuals with the RM genotype at the peak (Figure 6E) and a subsequent association analysis was performed on this subset of individuals. Permutation testing was performed in the data subset with 500 permutations, sufficient to estimate a 5% threshold. Based on the permutation result we used a significance threshold of 10^-3.82^, corresponding to a 5% FDR.

### Defining QTL/vQTL peaks

When a significant association was detected, we assigned SNPs in the same genomic regions to the same association peak as follows. First, the significant SNP with the lowest p-value was defined to be at the centre of the peak. Then, all SNPs within ±25 kb of this SNP was assigned to the same peak. This was performed for all significant SNPs in a sliding window fashion. Although slightly arbitrary it is nessesary to quantify the number of QTL rather than the absolute number of significant SNPs and 25kb was chosen based on the extend of observed distinct signals in the dataset.

 We further defined association peaks that mapped within 150kb of the position of the gene whose expression it affects as cis-QTL/vQTL, and all peaks located further away as trans-QTL/vQTL. The 150kb range was chosen on the observed placement of the majority of significant SNPs on the diagonal (Figures 1 and S1) and we intentionally err on the conservative side to ensure all trans-QTL/vQTL are true.

### Genome wide hotspots

We identified 19 areas in the genome where multiple phenotypes are affected by QTL or vQTL in close proximity (see Figures 1 and S1 and Table S1). The QTL/vQTL distribution throughout the genome is visually uneven. However, the borders of the hotspots are not very sensitive to different estimates and we included all regions that had an overrepresentation of QTL/vQTL for a number of phenotypes. The combined size of the hotspots is 22.3% of the whole genome. To assess that the chosen hotspots were enriched for QTL/vQTL we performed a chi-square test (i.e. ratio of QTL and vQTL within/outside the hotspot). We found a significant difference between the QTL/vQTL within vs. outside the hotspot (Table S3). 

### Evaluation of interactions

Gene-gene interactions (epistasis) can lead to a genetic variance–heterogeneity when the different alleles at the locus enhance or suppress the effects of other loci in the genome. Here, we studied the phenotypes for which significant vQTL were found in more detail to identify possible gene-gene interactions. First, genotype/phenotype maps were plotted for all possible two-locus combinations including an identified vQTL and other significant QTL or vQTL. These maps were manually evaluated to identify putative epistatic interactions. Candidate epistatic pairs were further explored by first identifying all genes located less than 10kb upstream or downstream from the QTL/vQTL peaks in the *Saccharomyces cerevisiae* reference genome (http://www.ncbi.nlm.nih.gov/genome/15/?project_id=128, Table S4). All pairs of genes in these 20kb regions were listed and used to mine the literature, the Yeastmine (http://yeastmine.yeastgenome.org/) and KEGG (http://www.genome.jp/kegg/) for previously described interactions between the gene-pairs or between the genes and the gene whose expression was analyzed. In this way, several putative functional interactions underlying the candidate epistatic pairs were identified.

### Data access

The expression data used in this study was obtained from: http://www.plosbiology.org/article/info%3Adoi%2F10.1371%2Fjournal.pbio.0060083 and is also available via Gene Expression Omnibus (GEO: http://www.ncbi.nlm.nih.gov/projects/geo/) accession number GSE9376 [9]. The genotypic data used in this was obtained from: http://blogs.ls.berkeley.edu/bremlab/data/ (see [7]).

## Supporting Information

Figure S1 **A per-chromosome distribution of QTL and vQTL with significant effects on gene-expression across the yeast genome (for a genome-wide visualization, see Figure 1).** Red and blue indicate significant QTL/vQTL peaks for glucose and ethanol treatments respectively. Closed circles indicate QTL affecting the mean level of expression and crosses vQTL affecting the variance heterogeneity. The eight solid grey lines are hotspots identified by [5]. The slanted red, blue and pink areas are hotspots identified by the study by [9], indicating glucose, ethanol and glucose-ethanol interaction respectively.
(TIFF)

Table S1 **Summary of the detected trans-QTL/vQTL per hotspot.**
(TXT)

Table S2 **All the peaks (QTL and vQTL for both treatments) per phenotype.**
(TXT)

Table S3 **Chi-square p-values for occurrence of QTL/vQTL in hotspots, per treatment, and test for difference between occurrence of vQTL and QTL in hotspots.**
(TXT)

Table S4 **Candidate genes per peak (for the phenotypes with significant vQTL only).**
(TXT)
