# Supplementary figures and images for: Variance Heterogeneity in Saccharomyces cerevisiae Expression Data: Trans-Regulation and Epistasis
Source: PLoS One. 2013 Nov 4;8(11):e79507. doi: 10.1371/journal.pone.0079507 (PMC3817098; doi:10.1371/journal.pone.0079507)

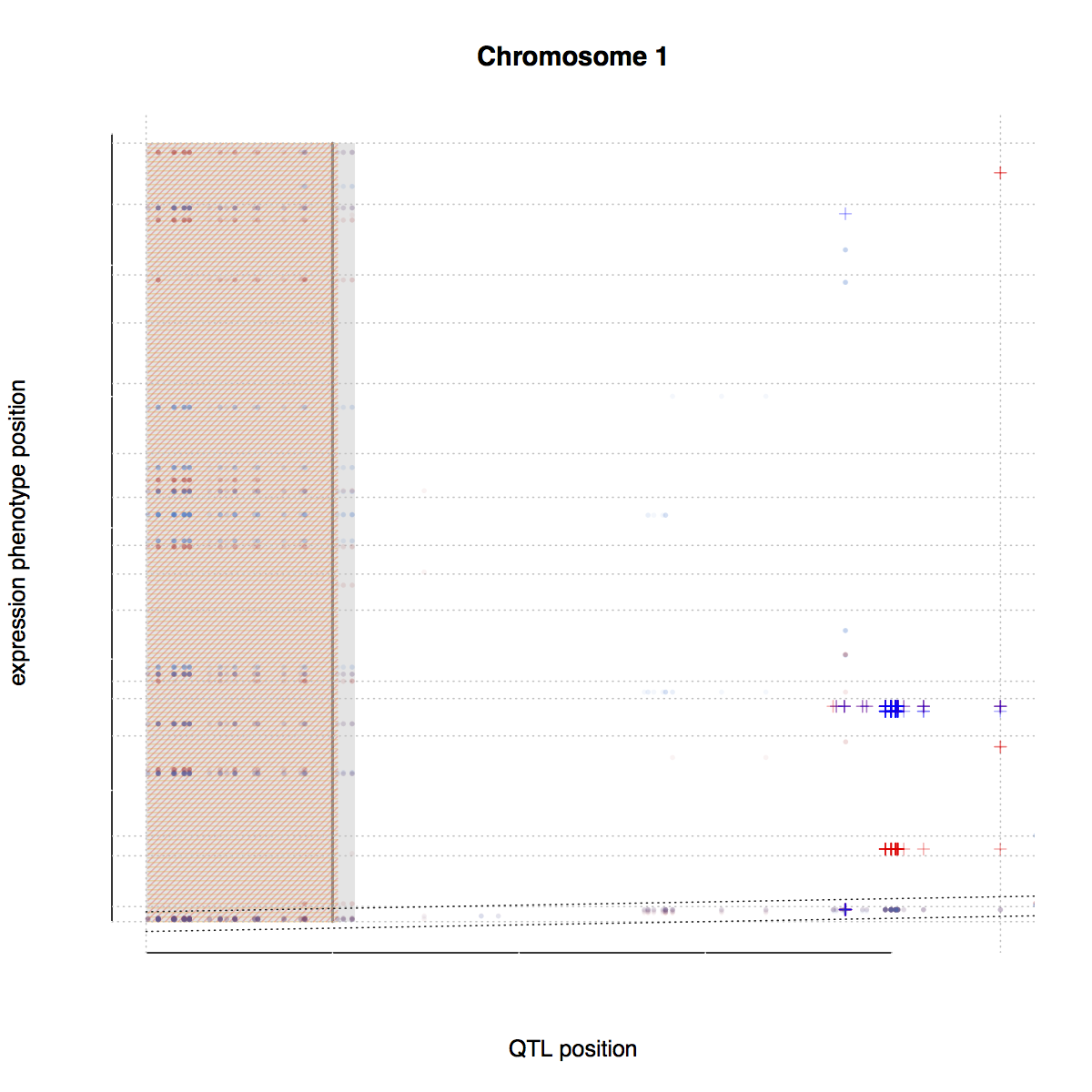

Supplement: Figure S1 — A per-chromosome distribution of QTL and vQTL with significant effects on gene-expression across the yeast genome (for a genome-wide visualization, see Figure 1). Red and blue indicate significant QTL/vQTL peaks for glucose and ethanol treatments respectively. Closed circles indicate QTL affecting the mean level of expression and crosses vQTL affecting the variance heterogeneity. The eight solid grey lines are hotspots identified by [5]. The slanted red, blue and pink areas are hotspots identified by the study by [9], indicating glucose, ethanol and glucose-ethanol interaction respectively. (TIFF) [file pone.0079507.s001.tiff]
